# The Effects of Air Pollution on COVID-19 Infection and Mortality—A Review on Recent Evidence

**DOI:** 10.3389/fpubh.2020.580057

**Published:** 2020-11-26

**Authors:** Nurshad Ali, Farjana Islam

**Affiliations:** Department of Biochemistry and Molecular Biology, Shahjalal University of Science and Technology, Sylhet, Bangladesh

**Keywords:** air pollution, COVID-19, infection, mortality, public health

## Abstract

The outbreak of COVID-19 has created a serious public health concern worldwide. Although, most of the regions around the globe have been affected by COVID-19 infections; some regions are more badly affected in terms of infections and fatality rates than others. The exact reasons for such variations are not clear yet. This review discussed the possible effects of air pollution on COVID-19 infections and mortality based on some recent evidence. The findings of most studies reviewed here demonstrate that both short-term and long-term exposure to air pollution especially PM_2.5_ and nitrogen dioxide (NO_2_) may contribute significantly to higher rates of COVID-19 infections and mortalities with a lesser extent also PM_10_. A significant correlation has been found between air pollution and COVID-19 infections and mortality in some countries in the world. The available data also indicate that exposure to air pollution may influence COVID-19 transmission. Moreover, exposure to air pollution may increase vulnerability and have harmful effects on the prognosis of patients affected by COVID-19 infections. Further research should be conducted considering some potential confounders such as age and pre-existing medical conditions along with exposure to NO_2_, PM_2.5_ and other air pollutants to confirm their detrimental effects on mortalities from COVID-19.

## Introduction

The outbreak of COVID-19 has created a global health crisis. Most of the countries in the world have been affected by COVID-19 infection. However, some regions have been more badly affected in terms of infections and fatality rates than others. These remarkable variations have raised significant questions related to the influence of air pollution to the extent of COVID-19 infections, and its mortality rate around the world. The exact reasons for this are not yet clear; some key aspects of the topic require further investigation.

It has been demonstrated that long-term exposure to air pollution is associated with an increased prevalence of respiratory diseases and deaths (1). Fine particulate matter with size <2.5 μm, PM_2.5_ is considered is one of the major health risk factors in the environment, causing millions of deaths annually around the world (2). The presence of PM_2.5_ and another one also PM_10_ are specifically associated with an increased rate of respiratory diseases, and of hospitalization for chronic lung disease and pneumonia (1, 2). Nitrogen dioxide (NO_2_) is another important air pollutant that is toxic to human respiratory systems when present at higher concentrations in the atmosphere It enters the atmosphere as a result of both anthropogenic and natural processes. As the outdoor anthropogenic sources, NO_2_ is mainly emitted from fuel combustion and transportation, in general, they come to the air from vehicle exhaust gases and domestic heating (3, 4). NO_2_ exerts adverse effects mainly on the respiratory system, however, prolonged exposure to NO_2_ is correlated with a wide range of severe illnesses such as hypertension, diabetes, and cardiovascular diseases and causes even death (2). An early study also showed that chronic exposure to NO_2_ causes cytokine-mediated inflammation in the lungs (5). Air pollution-related deaths include but are not limited to bronchitis, aggravated asthma, respiratory allergies, heart disease, and stroke (1, 2, 6).

Moreover, a link has been suggested between air pollution and infectious disease transmission (7). For example, bad air quality was associated with increased SARS fatality (8) as well as with increased incidence of influenza (9). In the laboratory environment, SARS-CoV-2 showed stability in ambient aerosols, which may be a considerable source of COVID-19 transmission (10). However, the association of ambient air pollutants with an increased incidence of COVID-19 in practical situation remains largely unknown. As a cofactor, atmospheric aerosol can induce indirect systemic effects in the human body and is associated with pro-inflammatory and oxidative mechanisms in the lungs, as well as altered immune system pathways (11).

The Setti et al. studies provided the first evidence that SARS-CoV-2 RNA can be present on outdoor PM in certain conditions of atmospheric stability and high levels of PM_10_, thus suggesting a possible application as an indicator of epidemic recurrence (12). A recent study by Pluchino et al. identified the PM_10_ concentration as an important factor of the risk in COVID-19 analysis (13). Although confounding effects may be present such as male gender, age, smoking and high population density as potential risk factors for higher morbidity and mortality of COVID-19 (11, 14). Therefore, caution has to be taken in translating high values of conventional indicators, such as PM_2.5_ and PM_10_ levels, into a straight measure of vulnerability. This review discussed the possible effects of air pollution on COVID-19 infection and mortality based on the recent data. PubMed, Google Scholar, Scopus, arXiv, and MedRxiv were searched up to September 30, 2020, to identify relevant publications using the following keywords: Air pollution and COVID-19 or SARS-CoV-2, Particulate matter (PM) and NO_2_ and COVID-19.

## Air Pollution and COVID-19 Infections and Mortality

Exposure to air pollution is considered as the major environmental cause of several diseases and premature death around the globe (15). Study evidence indicates that both short- and long-term exposures to air pollutants are associated with a wide range of adverse health effects (16, 17), such as higher fatality rates, increased hospital admissions and increased outpatient visits (18, 19). Some reviews highlighted the links between air pollution and COVID-19 (20–23). However, up to now a limited number of data-dependent studies have been conducted to investigate the association between air pollution and COVID-19 infection and mortality. The available studies that have demonstrated the effects of short-term (within 2 months of exposure) and long-term exposure (more than 2 months of exposure) to air pollution on COVID-19 infections and mortality are summarized in Table 1 and described below.

**Table 1:** Summary of the study findings related to air pollution effects on COVID-19.

**References**	**Study region**	**Study design**	**Period of study**	**Outcome**
Zhang et al. (24)	219 prefecture cities in China	Retrospective	January 24 to February 29, 2020	A positive correlation was observed between air pollution indicators and new COVID-19 confirmed cases. The SARS-CoV-2 spreading was between 5 and 7% when the air quality index (AQI) was increased by 10 units.
Zhu et al. (25)	120 cities in China	Retrospective	January 23 to February 29, 2020	A significant positive association was found for PM_2.5_, PM_10_, NO_2_, and O_3_ with newly COVID-19 confirmed cases. A 10-μg/m^3^ increase (lag0–14) in PM_2.5_, PM_10_, NO_2_, and O_3_ was associated with a 2.24, 1.76, 6.94, and 4.76% increase in the daily counts of COVID-19 cases, respectively.
Li et al. (7)	Wuhan and XiaoGan in China	Retrospective	January 26 to February 29, 2020.	A significant correlation was observed between COVID-19 incidence and AQI in both cities (*p* < 0.01). The incidence of COVID-19 was highly correlated with PM_2.5_ and NO_2_ in both cities.
Lin et al. (26)	29 Provinces in China	Retrospective	January 21 to April 3, 2020	Higher ambient CO concentration was a risk factor for the increased spreading of SARS-CoV-2, while higher temperatures, efficient ventilation and air pressure reduced its transmissibility.
Yao et al. (27)	Whuan in China	Retrospective	January 19 to March 15, 2020	After adjusting to temperature and relative humidity, SO_2_, NO_2_, CO, and O_3_, the case fatality rate (CFR) was positively associated with PM_2.5_ and PM_10_.
Jiang and Xu (28)	Whuan in China	Retrospective	January 25 to April 7, 2020	A significant positive correlation (*p* < 0.01) was observed between AQI especially PM_2.5_ and the daily COVID-19 deaths.
Pansini and Fornacca (14)	China, France, Germany, Iran, Italy, Spain, UK and the USA	Retrospective	NA	Increased SARS-CoV-2 infections were observed in the regions where high levels of PM_2.5_ and NO_2_ were present. A significant correlation was found between the levels of air quality with COVID-19 spread and mortality in six countries except for Spain and Germany.
Travaglio et al. (29)	England	Retrospective	As of April 10, 2020	The markers of poor air quality, such as NO and SO_2_ were associated with an increased rate of COVID-19 related deaths across England, after adjustment of population density.
Konstantinoudis et al. (30)	England	Retrospective	As of June 30, 2020	An increase of 0.5% and 1.4% in COVID-19 mortality rate was observed for every 1 μg/m3 increase in NO_2_ and PM_2.5_, respectively.
Magazzino et al. (31)	3 cities in France	Retrospective	NA	This study showed a direct relationship between air pollutants (PM_2.5_ and PM_10_) and COVID-19 fatality.
Ogen (32)	France, Germany, Italy, Spain	Retrospective	January to February 2020	About 78% of deaths occurred in just five regions of northern Italy and central Spain, where NO_2_ were present at the highest concentrations combined with downward air pressure.
Mele and Magazzino (33)	25 cities in India	Retrospective	January 29 to May 18, 2020	In machine learning (ML) analysis with Causal Direction from Dependency (D2C) algorithm, a direct relationship was found between the concentration of PM_2.5_ and COVID-19 mortality.
Zoran et al. (34)	Milan, Italy	Retrospective	January 1 to April 30, 2020	COVID-19 infections showed a positive correlation with ground level O_3_. However, ground level NO_2_ was inversely correlated with COVID-19 infections. Outdoor airborne aerosols might be the possible carriers of COVID-19 transmission.
Zoran et al. (35)	Milan, Italy	Retrospective	January 1 to April 30, 2020	Daily new cases of COVID-19 were positively related to PM and AQI. Dry air supports SARS-CoV-2 transmission. Warm-season may not have a role in spreading viral infection.
Fattorini and Regoli (36)	Italy	Retrospective	As of April 27, 2020	Long-term air-quality data showed a significant correlation with COVID-19 cases in 71 provinces in Italy, provided further evidence that chronic exposure to air pollution may influence the viral spreads.
Setti et al. (37)	110 Provinces in Italy	Retrospective	February 24 to March 13, 2020	A significant association has been observed between the geographical distribution of daily PM_10_ exceedances and the initial spreading of COVID-19 in the Italian provinces.
Coker et al. (38)	Northern Italy	Retrospective	January 1 to April 30, 2020	A positive association was observed between ambient PM_2.5_ concentration and excess COVID-19 related mortality. A one-unit increase in PM_2.5_ concentration (μg/m3) was associated with a 9% increase in the COVID-19 related fatality.
Frontera et al. (39)	Italy	Retrospective	As of March 31, 2020	A high number of COVID-19 cases were found in the most polluted regions and the affected patients required ICU admission. The mortality was two-fold higher in these polluted regions than the other regions.
Coccia (40)	Northern Italy	Retrospective	As of March 17, to April 2020	An association was observed between accelerating and vast diffusion of COVID-19 and air pollution. This study demonstrated that contaminated air accelerates the transmission of the SARS-CoV-2 to humans other than the transmission from human to human.
Vasquez-Apestegui et al. (41)	20 districts in Lima (Peru)	Retrospective	As of June 12, 2020	The higher rates of spread of COVID-19 in Lima were associated with the previous long-term PM_2.5_ exposure.
Andree BPJ (42)	355 municipalities in the Netherlands	Retrospective	As of March 31, 2020	PM_2.5_ was a highly significant predictor of COVID-19 cases and the related hospital admissions. It was also observed that COVID-19 cases were increased by almost 100% when pollutant concentrations were increased by 20%.
Hendryx and Luo (43)	USA	Retrospective	As of May 31, 2020	In regression analyses, COVID-19 prevalence and mortality rates were significantly associated with greater diesel particulate matter (DPM).
Wu et al. (44)	3000 counties in the U.S.A.	Cross-sectional	As of April 04, 2020	An increase of only 1 μg/m^3^ in long-term PM_2.5_ exposure is associated with an 8% increase in the COVID-19 fatality rate.
Adhikari and Yin (45)	New York, USA	Retrospective	March 1 to April 20, 2020	Short-term exposures to ozone and other meteorological factors could be associated with COVID-19 transmission and initiation of the disease, but disease aggravation and fatality depend on other factors.
Bashir et al. (46)	California, USA	Retrospective	March 4 to April 24, 2020	Air pollutants such as PM_10_, PM_2.5_, SO_2_, NO_2_, and CO showed a significant correlation with the COVID-19 epidemic.

## Short-Term Exposure to Air Pollution and COVID-19

A recent study has examined the geographical properties of the COVID-19 infection and associated it with various annual satellite and ground level of air quality index in eight countries including Italy, Spain, Germany, France, UK, USA, Iran and China and found more viral infections in the regions where high levels of PM_2.5_ and NO_2_ were present (14). The study observed a significant correlation between the levels of air quality and COVID-19 spread and mortality in six countries except for Spain and Germany. Of these countries, Italy showed the strongest correlations in terms of both for infection and mortality, while population size and density did not correlate with COVID-19 incidence. In China, population density showed a similar positive correlation between infection and mortality than to air pollution, while in the UK and USA, population density had a stronger correlation with infection and mortality than air pollution. On the other hand, Iran showed a significant relationship with NO_2_ distribution than air population variables. In Spain, levels of air pollution could not explain the rate of infections and mortality; however, population size and density showed a negative correlation with the infections. In Germany, population density showed a weak correlation with infections while particulate matters showed a weak negative correlation. The authors noted that the negative association between virus infection and population density, perhaps because of the huge movement of people from large cities to the countryside taking the virus with them (14). Another retrospective study by Li et al. showed a significant correlation between air quality index (AQI) and incidence of COVID-19 in Wuhan (*p* < 0.05) and Xiao Gan (*p* < 0.01) in China (7). The authors noted that among four ambient air pollutants (PM_2.5_, PM_10_, NO_2_, and CO), PM_2.5_ and NO_2_ were strongly correlated with the incidence of COVID-19. Moreover, from the metrological parameter, the only temperature showed a consistent correlation with COVID-19 incidence in both cities (7). Another investigation reported a positive correlation between air pollution indicators and new COVID-19 confirmed cases in China (24). The SARS-CoV-2 spreading was between 5 and 7% when AQI was increased by 10 units (24). A significant positive association was found for PM_2.5_, PM_10_, NO_2_, and O_3_ with newly COVID-19 confirmed cases in 120 cities in China (25). A 10-μg/m^3^ increase (lag0–14) in PM_2.5_, PM_10_, NO_2_, and O_3_ was associated with a 2.24, 1.76, 6.94, and 4.76% increase in the daily counts of COVID-19 cases, respectively (25). Lin et al. reported that higher ambient CO concentration was a risk factor for the increased spreading of SARS-CoV-2, while higher temperature, efficient ventilation and air pressure reduced its transmissibility (26). In another study, after adjusting to temperature and relative humidity, SO_2_, NO_2_, CO, and O_3_, the case fatality rate (CFR) showed a positive association with PM_2.5_ and PM_10_ in China (27). A significant positive correlation (*p* < 0.01) was also observed between AQI especially PM_2.5_ and the daily COVID-19 deaths in Wuhan, China (28). In three cities in France, a direct relationship was found between air pollutants (PM_2.5_ and PM_10_) and COVID-19 fatality (31). It is also plausible that SARS-CoV-2 transmission by fomites and aerosol, and the viral particle can remain infectious and viable in aerosol for several hours and on surfaces for several days (10).

A study collecting data from 110 Italian cities, reported a significant association between the geographical distribution of daily PM_10_ exceedances and the initial spreading of COVID-19 (37). It has been suggested that particulate matter (PM_10_) may serve as a carrier for droplet nuclei, increasing the spread of SARS-CoV-2 (37). This result was supported by another study that showed an association between accelerate and vast diffusion of COVID-19 and air pollution (40). Moreover, this study demonstrated that contaminated air accelerates the transmission of the SARS-CoV-2 to humans other than the transmission from human to human. Another study in Italy found a high number of COVID-19 cases were in the most polluted regions and the affected patients required ICU admission and the mortality was two-fold higher in these polluted regions than the other regions (39). In the USA, two retrospective studies also determined the effects of air pollutants on COVID-19. Adhikari and Yin demonstrated that short-term exposures to ozone and other meteorological factors could be associated with COVID-19 transmission and initiation of the disease, but disease aggravation and fatality depend on other factors (45). In other study air pollutants such as PM_10_, PM_2.5_, SO_2_, NO_2_, and CO showed a significant correlation with the COVID-19 epidemic (46).

## Long-Term Exposure to Air Pollution and COVID-19

A new study looked at COVID-19 mortalities in four countries in Europe that have been most affected by the novel virus-Spain, Italy, France, and Germany (32). It was found that about 78% of deaths occurred in just five regions of northern Italy and central Spain, where NO_2_ were present at the highest concentrations combined with downward air pressure, which prevented the dispersion of air pollutants. The author demonstrated that prolonged exposure to NO_2_ may contribute to mortality caused by the COVID-19 infection in these areas. Another study used the data from US Environmental Protection Agency Environmental Justice Screen (EPAEJS) and prevalence and fatality rates as of 31 may 2020 demonstrated that, after adjusting for covariates, COVID-19 incidence and mortality rates were significantly correlated with greater diesel particulate matter (DPM) (43). In Italy, a recent study also reported a correlation between COVID-19 mortality in northern Italy where the high levels of pollutants were present (47). Long-term air-quality data showed a significant correlation with COVID-19 cases in 71 provinces in Italy, providing further indication that chronic exposure to air pollution may influence the viral spreads (36).

A recent study collecting data from 355 municipalities in the Netherlands showed PM_2.5_ as a highly significant predictor of COVID-19 cases and hospital admissions (42). It also observed that COVID-19 cases were increased by almost 100% when pollutant concentrations were increased by 20%. A study in England reported an increase of 0.5 and 1.4% in the COVID-19 mortality rate for every 1 μg/m3 increase in NO_2_ and PM_2·5_, respectively after adjusting of confounders (30). This study evidence of an effect of long-term NO_2_ exposure on COVID-19 mortality, while the effect of PM_2·5_ remains unclear. Another recent study also provided evidence on the association of air pollution with SARS-CoV-2 lethality in England (29). The authors showed an association between fossil fuels released pollutants and vulnerability to viral infection (29). This finding suggests that people exposed to chronic greater levels of air pollution might be more vulnerable to viral infection. According to the UK's air quality and emissions news and information site, in France, where COVID-19 distribution maps depicted areas with a very large number of severely COVID-19 affected patients required hospitalization (https://airqualitynews.com/2020/04/09/why-air-pollution-is-linked-to-a-faster-spread-of-coronavirus/). A similar trend has also been observed in the Czech Republic where a higher number of people have been diagnosed with COVID-19 in intensely polluted areas of Prague. Furthermore, a nationwide study in the USA demonstrated a significant association between long-term exposure to PM_2.5_ and the risk of deaths from COVID-19 (44). This study showed that an increase of only 1 μg/m^3^ in PM_2.5_ exposure is associated with an 8% increase in the COVID-19 fatality rate and long-term exposure to air pollution largely increases the COVID-19 mortality rate (44).

A study collected data from 25 cities in India, reported a direct relationship between the concentration of PM_2.5_ and COVID-19 mortality (33). In Italy, a positive correlation of PM, AQI and ground-level O_3_ was observed with COVID-19 infections (34, 35). The authors also demonstrated that outdoor airborne aerosols might be the possible carriers of COVID-19 transmission (34). Dry air supports SARS-CoV-2 transmission. Warm-season may not have a role in spreading viral infection (35). Another study in Italy reported a positive association between ambient PM_2.5_ concentration and excess COVID-19 related mortality (38). A one-unit increase in PM_2.5_ concentration (μg/m3) was associated with a 9% increase in the COVID-19 related fatality (38). In Peru, the higher rates of spread of COVID-19 in Lima were associated with the previous long-term PM_2.5_ exposure (41). Currently, the Center for Research on Energy and Clean Air (CREA) reported that greater levels of air pollution interfere with the body's normal defenses against airborne viruses including SARS-CoV-2 (48). The agency also added that air pollution increases the risk of hospitalization and death from COVID-19.

The major limitations of most of the studies (both short-term and long-term) are that correlation between air pollution and COVID-19 incidence and mortality have been made without adjustment for population size, age distribution or other confounding variables in the analysis. The increased infection and mortality rates in polluted areas might be also associated with another immune defense system. The compromised immune defense response because of air pollution has been observed in patients with severe pneumonia during previous pandemics (8, 49). Recent data also suggest that higher mortality from COVID-19 may also be associated with cytokine storm syndrome (50). However, all the above study findings should be interpreted more cautiously as the virus infection is still ongoing in many countries. Nevertheless, the larger the geographic regions are affected by the COVID-19 pandemic, the small regional factors will play a role (14). Besides, air pollutants, a positive correlation was also observed for the faster spread of COVID-19 with low temperature, lung cancer prevalence, smoking, low vitamin D levels, and UV index (51, 52). Therefore, besides the plausible mechanisms of airborne transmission, other transmission routes of the virus in humans need to be considered while interpreting the impact of air quality on the virus spread. It is also important to mention that findings from the recent studies are not new to highlight the substantial link between levels of air pollution and deaths from viral diseases. A previous study showed that patients affected by SARS, a virus similar to COVID-19, were about 84% more likely to die if they lived in a highly polluted area over time (8). Most of the available data indicate that COVID-19 infections and fatality rates are more frequent in highly polluted regions than elsewhere. It has also been observed that because of lockdown strategies, air pollution was reduced in some regions in India and China (53, 54). So maintaining air quality may be an important and effective approach to prevent COVID-19 transmission.

## Conclusions and Recommendation

In conclusion, exposure to air pollution especially NO_2_ and PM_2.5_ may increase the susceptibility of infection and mortality from COVID-19. The available data also indicate that exposure to air pollution may influence COVID-19 transmission. Moreover, air pollution can cause adverse effects on the prognosis of patients affected by SARS-CoV-2 infection. The available research findings on this topic may help the epidemiologists to select a proper measure to prevent such an outbreak in the future. Attention should also be paid to the poor communities, who are susceptible to be exposed to indoor air pollution, contributing to a greater risk of becoming severely ill from COVID-19 infections. Air quality should be counted as an important part of an integrated approach toward public health protection and prevention to the spread of epidemics. Further research should be conducted focusing on additional confounders such as age and pre-existing medical conditions along with prolonged exposure to NO_2_, PM_2.5_, and other air pollutants to confirm their detrimental effects on mortalities from COVID-19.

## Author Contributions

NA wrote and revised the manuscript. FI helped in writing and revision of the manuscript. All authors contributed to the article and approved the submitted version.

## Conflict of Interest

The authors declare that the research was conducted in the absence of any commercial or financial relationships that could be construed as a potential conflict of interest.
